# Supplementary material for: Assessing the Hypertension Risk: A Deep Dive into Cereal Consumption and Cooking Methods—Insights from China
Source: Nutrients. 2024 Sep 8;16(17):3027. doi: 10.3390/nu16173027 (PMC11397540; doi:10.3390/nu16173027)
Supplement: Supplementary file 1 [file nutrients-16-03027-s001.zip › nutrients-3136063-supplementary.pdf]

Supplementary Material

Assessing the Hypertension Risk: A Deep Dive into Cereal Consumption and Cooking Methods—Insights from China

Table of Contents

Supplementary Figure S1. Flow chart of participants enrolled in CHNS 1997–2015.

Supplementary Figure S2. Hazard ratios (95% CIs) of hypertension for consumption of total cereals in subgroup analyses in CHNS 1997–2015.

Supplementary Figure S3. Hazard ratios (95% CIs) of hypertension for consumption of cereals with different cooking methods in subgroup analyses in CHNS 1997–2015.

Supplementary Table S1. The primary cereal products within each cooking method group in CHNS 1997–2015.

Supplementary Table S2. Hazard ratios (95% CIs) for hypertension risk for consumption of refined grains in CHNS 1997–2015.

Supplementary Table S3. Hazard ratios (95% CIs) for hypertension risk for consumption of cereals in subgroup analyses in CHNS 1997–2015.

Supplementary Table S4. Hazard ratios (95% CIs) for hypertension risk for consumption of cereals in sensitivity analyses in CHNS 1997–2015.

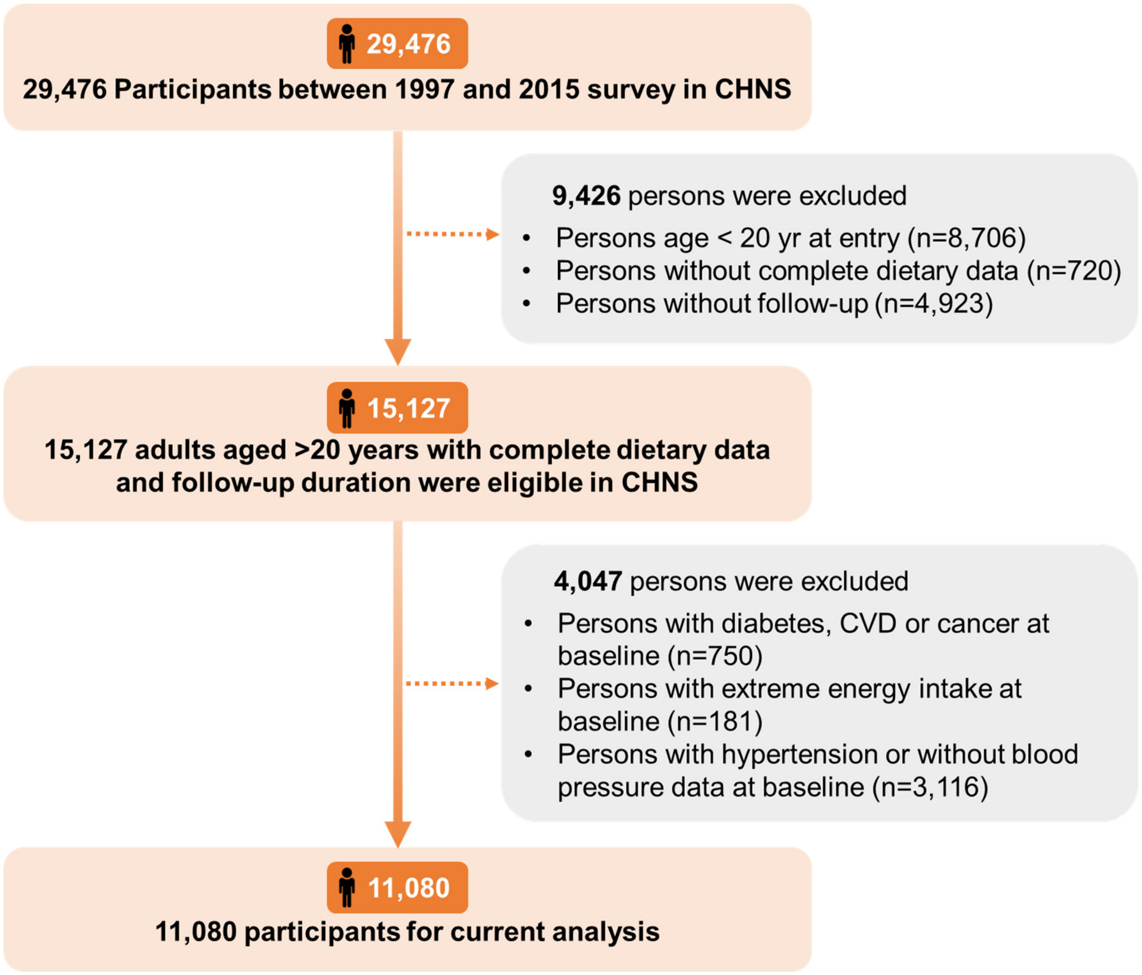

Supplementary Figure S1. Flow chart of participants enrolled in CHNS 1997–2015.

Total cereal intake

Subgroup	HR (95% CIs)	*P* for interaction*
**Age**		
< 60 yr	1.06 (1.02–1.12)	0.369
≥ 60 yr	0.95 (0.85–1.06)	
**Sex**		
Men	1.01 (0.95–1.08)	0.012
Women	1.06 (1.00–1.13)	
**Smoking status**		
Never	1.08 (1.02–1.13)	0.001
Former or current	0.96 (0.88–1.04)	
**Alcohol drinking**		
Abstainer	1.07 (1.01–1.13)	0.150
Drinker	0.99 (0.92–1.07)	
**BMI (kg/m^2)**		
< 24	1.05 (1.00–1.11)	0.397
≥ 24	1.02 (0.94–1.10)	
**Physical activity**		
Low	1.02 (0.95–1.09)	0.806
Moderate to vigorous	1.04 (0.97–1.10)	
**Education**		
Below great high school	1.04 (0.99–1.08)	0.500
Great high school and above	1.09 (0.93–1.27)	
**Household income**		
Below median	1.06 (1.00–1.13)	0.194
Above median	1.03 (0.97–1.10)	

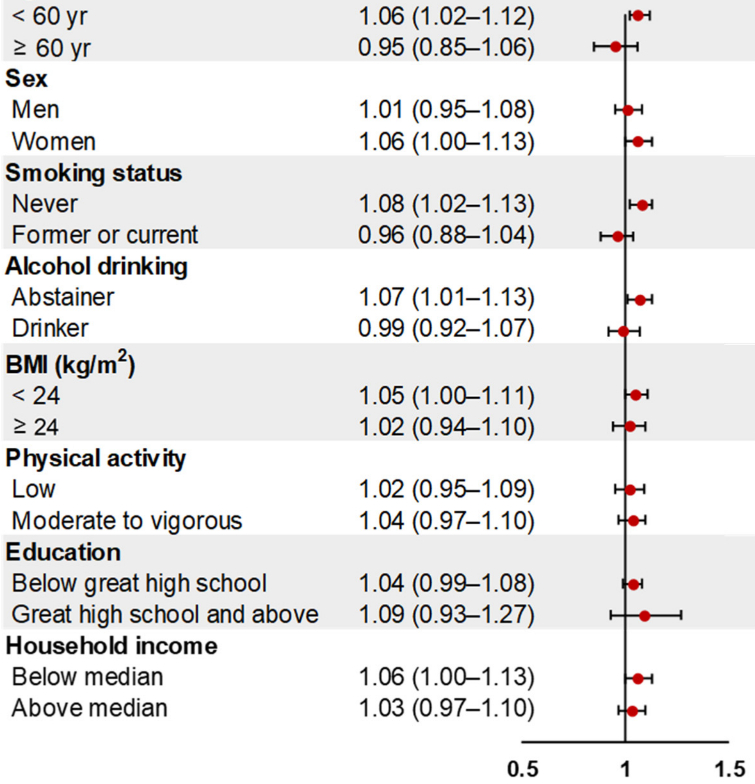

Supplementary Figure S2. Hazard ratios (95% CIs) of hypertension for consumption of total cereals in subgroup analyses in CHNS 1997–2015.

HRs and 95% CIs were estimated using Cox proportional hazard regression models adjusted for age, sex, nationality (Han or non-Han), marital status (never married, married or living as married, widowed/divorced/separated, or unknown), BMI, household income (quintile), urbanization index, education (less than high school, high school, some college or at least college), physical activity (no regular activity, low to moderate activity or vigorous activity), smoking (never, former, current, or unknown), alcohol drinking status (abstainer or drinker) and medical insurance, total energy intake, vegetable intake, fruit intake, total meat intake, sodium intake, and potassium intake. CI, confidence interval; HR, Hazard ratio.

* *P* values for interaction were calculated by likelihood–ratio tests comparing Cox proportional-hazards models with and without cross-product terms for each level of baseline stratifying variables.

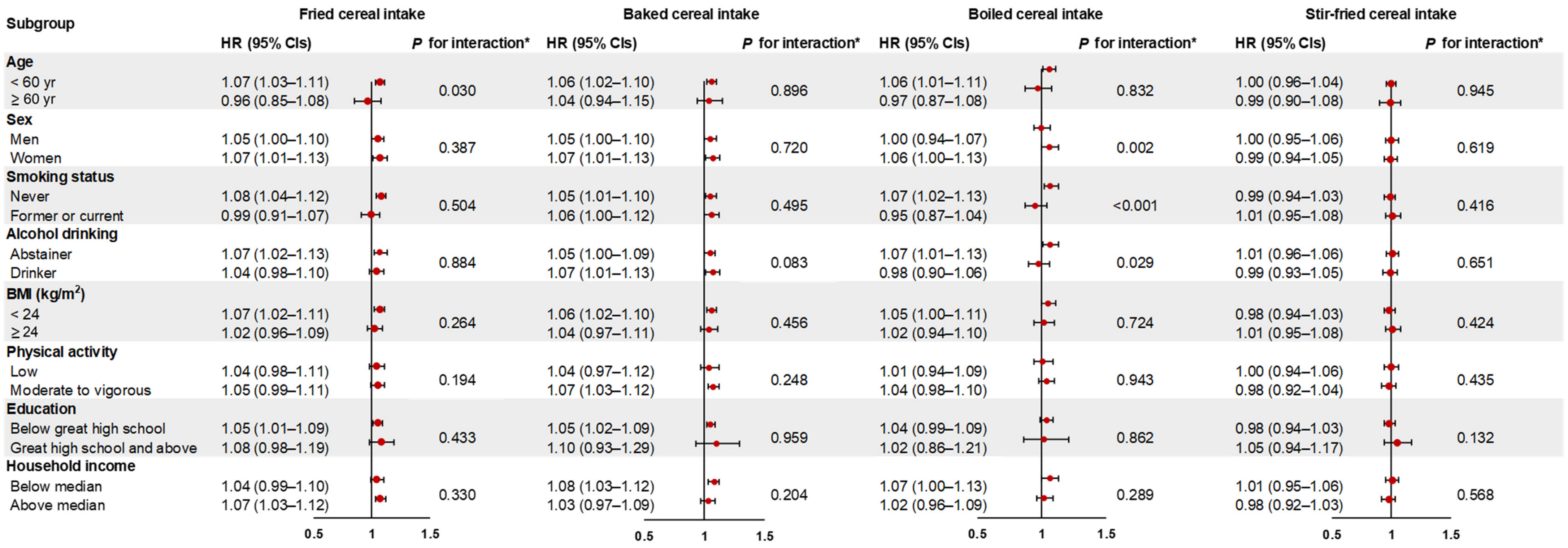

Supplementary Figure S3. Hazard ratios (95% CIs) of hypertension for consumption of cereals with different cooking methods in subgroup analyses in CHNS 1997–2015.

HRs and 95% CIs were estimated using Cox proportional hazard regression models adjusted for age, sex, nationality (Han or non-Han), marital status (never married, married or living as married, widowed/divorced/separated, or unknown), BMI, household income (quintile), urbanization index, education (less than high school, high school, some college or at least college), physical activity (no regular activity, low to moderate activity or vigorous activity), smoking (never, former, current, or unknown), alcohol drinking status (abstainer or drinker) and medical insurance, total energy intake, vegetable intake, fruit intake, total meat intake, sodium intake, and potassium intake. CI, confidence interval; HR, Hazard ratio.

* *P* values for interaction were calculated by likelihood-ratio tests comparing Cox proportional-hazards models with and without cross-product terms for each level of baseline stratifying variables.

Supplementary Table S1 The primary cereal products within each cooking method group in the CHNS 1997–2015*.

**Cooking groups**	**Main cereal products***
Fried cereals	Wheat, rice, corn, millet and yellow millet, barley, others
Baked cereals	Wheat, corn, rice, millet and yellow millet, barley, others
Boiled cereals	Rice, wheat, corn, millet and yellow millet, barley, others
Stir-fried cereals	Rice, wheat, corn, millet and yellow millet, barley, others

*The primary cereal products within each cooking method group were evaluated through the Chinese FCT.

Supplementary Table S2 Hazard ratios (95% CIs) for hypertension risk for consumption of refined grains in CHNS 1997–2015.

	Dietary refined grain intake (g/2000 kcal/day)				
	**Q1**	**Q2**	**Q3**	**Q4**	**P-trend**
**Total refined grains**					
Median (g/2000 kcal/day)	238	317	395	556	
Cases/person-years	865 /19,390	963 /24,930	961 /24,930	854 /16,620	
Model 1*	1	0.96 (0.88–1.06)	0.95 (0.87–1.04)	1.01 (0.92–1.12)	0.892
Model 2†	1	0.99 (0.90–1.09)	0.99 (0.90–1.09)	1.04 (0.95–1.15)	0.437
Model 3‡	1	1.01 (0.92–1.12)	1.03 (0.92–1.15)	1.10 (0.96–1.26)	0.193
**Fried refined grains**					
Median (g/2000 kcal/day)	0	6	15	38	
Cases/person-years	2,449 /55,867	473 /12,396	414 /9,297	307 /6,198	
Model 1*	1	1.13 (1.02–1.24)	1.31 (1.18–1.46)	1.29 (1.14–1.45)	<.0001
Model 2†	1	1.06 (0.96–1.17)	1.24 (1.12–1.38)	1.14 (1.01–1.29)	<0.001
Model 3‡	1	1.04 (0.94–1.15)	1.21 (1.08–1.35)	1.10 (0.96–1.24)	0.006
**Baked refined grains**					
Median (g/2000 kcal/day)	0	8	22	67	
Cases/person-years	2,414 /55,741	450 /11,429	438 /9,351	341 /6,234	

Model 1*	1	1.14 (1.03–1.26)	1.46 (1.32–1.62)	1.25 (1.12–1.40)	<0.001
Model 2†	1	1.09 (0.99–1.21)	1.37 (1.24–1.52)	1.15 (1.02–1.29)	<0.001
Model 3‡	1	1.09 (0.98–1.21)	1.39 (1.24–1.55)	1.16 (1.02–1.31)	<0.001
**Boiled refined grains**					
Median (g/2000 kcal/day)	206	290	367	519	
Cases/person-years	867 /19,390	945 /24,930	948 /24,930	883 /16,620	
Model 1*	1	0.90 (0.82–0.99)	0.90 (0.82–0.99)	0.97 (0.88–1.07)	0.537
Model 2†	1	0.95 (0.86–1.04)	0.96 (0.87–1.05)	1.03 (0.93–1.14)	0.528
Model 3‡	1	1.02 (0.92–1.13)	1.07 (0.95–1.19)	1.20 (1.05–1.37)	0.009
**Stir-fried refined grains**					
Median (g/2000 kcal/day)	0	6	15	39	
Cases/person-years	2,090 /41,790	630 /16,452	542 /12,348	381 /8,232	
Model 1*	1	0.98 (0.90–1.07)	1.03 (0.93–1.13)	0.91 (0.82–1.02)	0.307
Model 2†	1	0.99 (0.91–1.09)	1.06 (0.96–1.16)	0.95 (0.85–1.06)	0.840
Model 3‡	1	0.99 (0.90–1.08)	1.07 (0.97–1.18)	0.97 (0.87–1.08)	0.836

Time-dependent Cox proportional hazard regression models were used to assess HRs (95% CIs) of hypertension. CI, confidence interval; HR, hazard ratio.

* Model 1 was adjusted for age and sex.

† Model 2 was adjusted for model 1 plus nationality (Han or non-Han), marital status (never married, married or living as married, widowed/divorced/separated, or unknown), BMI, household income (quintile), urbanization index, education (less than high school, high school, some college or at least college), physical activity (no regular activity, low to moderate activity or vigorous activity), smoking (never, former, current, or unknown), alcohol drinking status (abstainer or drinker) and medical insurance.

‡ Model 3 was adjusted for model 2 plus total energy intake, vegetable intake, fruit intake, total meat intake, sodium intake, and potassium intake.

Supplementary Table S3. Hazard ratios (95% CIs) for hypertension risk for consumption of cereals in subgroup analyses in CHNS 1997–2015.

		Total cereal intake		Fried cereal intake		Baked cereal intake		Boiled cereal intake		Stir-fried cereal intake	
**Subgroup**	**Case/N**	**HR (95% CIs)**	**P interaction ***	**HR (95% CIs)**	**P interaction ***	**HR (95% CIs)**	**P interaction ***	**HR (95% CIs)**	**P interaction ***	**HR (95% CIs)**	**P interaction ***
**Age**											
<60 yr	3,069/9,997	1.06 (1.02–1.12)	0.369	1.07 (1.03–1.11)	0.030	1.06 (1.02–1.10)	0.896	1.06 (1.01–1.11)	0.832	1.00 (0.96–1.04)	0.945
≥60 yr	574/1,083	0.95 (0.85–1.06)		0.96 (0.85–1.08)		1.04 (0.94–1.15)		0.97 (0.87–1.08)		0.99 (0.90–1.08)	
**Sex**											
Men	1,825/4,938	1.01 (0.95–1.08)	0.012	1.05 (1.00–1.10)	0.387	1.05 (1.00–1.10)	0.720	1.00 (0.94–1.07)	0.002	1.00 (0.95–1.06)	0.619
Women	1,818/6,142	1.06 (1.00–1.13)		1.07 (1.01–1.13)		1.07 (1.01–1.13)		1.06 (1.00–1.13)		0.99 (0.94–1.05)	
**Smoking status**											
Never	2,435/7,920	1.08 (1.02–1.13)	0.001	1.08 (1.04–1.12)	0.504	1.05 (1.01–1.10)	0.495	1.07 (1.02–1.13)	<0.001	0.99 (0.94–1.03)	0.416
Former or current	1,208/3,160	0.96 (0.88–1.04)		0.99 (0.91–1.07)		1.06 (1.00–1.12)		0.95 (0.87–1.04)		1.01 (0.95–1.08)	
**Alcohol drinking**											
Abstainer	2,276/7,340	1.07 (1.01–1.13)	0.150	1.07 (1.02–1.13)	0.884	1.05 (1.00–1.09)	0.083	1.07 (1.01–1.13)	0.029	1.01 (0.96–1.06)	0.651
Drinker	1,367/3,740	0.99 (0.92–1.07)		1.04 (0.98–1.10)		1.07 (1.01–1.13)		0.98 (0.90–1.06)		0.99 (0.93–1.05)	
**BMI (kg/m^2)**											
<24	2,536/8,334	1.05 (1.00–1.11)	0.397	1.07 (1.02–1.11)	0.264	1.06 (1.02–1.10)	0.456	1.05 (1.00–1.11)	0.724	0.98 (0.94–1.03)	0.424
≥24	1,107/2,746	1.02 (0.94–1.10)		1.02 (0.96–1.09)		1.04 (0.97–1.11)		1.02 (0.94–1.10)		1.01 (0.95–1.08)	
**Physical activity**											
Low	1,287/4,632	1.02 (0.95–1.09)	0.806	1.04 (0.98–1.11)	0.194	1.04 (0.97–1.12)	0.248	1.01 (0.94–1.09)	0.943	1.00 (0.94–1.06)	0.435
Moderate to vigorous	2,045/5,598	1.04 (0.97–1.10)		1.05 (0.99–1.11)		1.07 (1.03–1.12)		1.04 (0.98–1.10)		0.98 (0.92–1.04)	
**Education**											
Below great high school	3,329/9,518	1.04 (0.99–1.08)	0.500	1.05 (1.01–1.09)	0.433	1.05 (1.02–1.09)	0.959	1.04 (0.99–1.09)	0.862	0.98 (0.94–1.03)	0.132
Great high school and above	314/1,562	1.09 (0.93–1.27)		1.08 (0.98–1.19)		1.10 (0.93–1.29)		1.02 (0.86–1.21)		1.05 (0.94–1.17)	
**Household income**											
Below median	2,147/5,490	1.06 (1.00–1.13)	0.194	1.04 (0.99–1.10)	0.330	1.08 (1.03–1.12)	0.204	1.07 (1.00–1.13)	0.289	1.01 (0.95–1.06)	0.568
Above median	1,461/5,492	1.03 (0.97–1.10)		1.07 (1.03–1.12)		1.03 (0.97–1.09)		1.02 (0.96–1.09)		0.98 (0.92–1.03)	

HRs and 95% CIs were estimated using Cox proportional hazard regression models adjusted for age, sex, nationality (Han or non-Han), marital status (never married, married or living as married, widowed/divorced/separated, or unknown), BMI, household income (quintile), urbanization index, education (less than high school, high

school, some college or at least college), physical activity (no regular activity, low to moderate activity or vigorous activity), smoking (never, former, current, or unknown), alcohol drinking status (abstainer or drinker) and medical insurance, total energy intake, vegetable intake, fruit intake, total meat intake, sodium intake, and potassium intake. CI, confidence interval; HR, Hazard ratio.

* *P* values for interaction were calculated by likelihood-ratio tests comparing Cox proportional–hazards models with and without cross-product terms for each level of baseline stratifying variables.

Supplementary Table S4. Hazard ratios (95% CIs) for hypertension risk for consumption of cereals in sensitivity analyses in CHNS 1997–2015.

	Dietary cereal intake (g/2000 kcal/day)				
	Q1	Q2	Q3	Q4	P-trend
**Further adjusting healthy insurance**					
Total cereal	1	1.05 (0.95–1.16)	1.08 (0.97–1.20)	1.16 (1.04–1.30)	0.011
Fried cereal	1	1.04 (0.94–1.16)	1.11 (0.99–1.24)	1.20 (1.06–1.36)	0.002
Baked cereal	1	1.08 (0.97–1.20)	1.34 (1.20–1.50)	1.21 (1.07–1.37)	<0.001
Boiled cereal	1	0.98 (0.89–1.08)	1.04 (0.94–1.16)	1.11 (0.99–1.25)	0.034
Stir-fried cereal	1	0.95 (0.87–1.04)	1.05 (0.96–1.16)	0.99 (0.89–1.11)	0.731
**Further adjusting AHEI**					
Total cereal	1	1.06 (0.96–1.17)	1.08 (0.97–1.20)	1.17 (1.04–1.31)	0.009
Fried cereal	1	1.02 (0.92–1.13)	1.08 (0.96–1.20)	1.16 (1.02–1.31)	0.018
Baked cereal	1	1.04 (0.94–1.16)	1.30 (1.16–1.45)	1.17 (1.03–1.33)	<0.001
Boiled cereal	1	0.98 (0.89–1.08)	1.05 (0.94–1.17)	1.13 (1.00–1.26)	0.023
Stir-fried cereal	1	0.95 (0.86–1.04)	1.05 (0.95–1.15)	1.00 (0.90–1.11)	0.692
**Further adjusting SSB intake**					
Total cereal	1	1.05 (0.96–1.16)	1.08 (0.97–1.20)	1.16 (1.03–1.30)	0.014
Fried cereal	1	1.05 (0.95–1.17)	1.11 (1.00–1.24)	1.20 (1.06–1.36)	0.002
Baked cereal	1	1.08 (0.97–1.20)	1.34 (1.20–1.50)	1.20 (1.06–1.36)	<0.001
Boiled cereal	1	0.98 (0.89–1.09)	1.04 (0.94–1.16)	1.11 (0.99–1.25)	0.039
Stir-fried cereal	1	0.96 (0.88–1.05)	1.06 (0.97–1.17)	1.00 (0.90–1.12)	0.591
**Excluding participants with hypertension at first 2 years**					
Total cereal	1	1.06 (0.96–1.17)	1.06 (0.95–1.18)	1.14 (1.01–1.28)	0.045
Fried cereal	1	1.05 (0.95–1.17)	1.11 (0.99–1.24)	1.22 (1.07–1.39)	0.002
Baked cereal	1	1.07 (0.96–1.19)	1.32 (1.18–1.48)	1.19 (1.04–1.35)	<0.001

Boiled cereal	1	0.97 (0.88–1.08)	1.02 (0.91–1.14)	1.09 (0.97–1.23)	0.100
Stir-fried cereal	1	0.96 (0.88–1.06)	1.04 (0.95–1.15)	0.98 (0.88–1.10)	0.925
**Excluding participants with incomplete covariate data**					
Total cereal	1	1.02 (0.91–1.14)	1.04 (0.93–1.17)	1.11 (0.98–1.26)	0.105
Fried cereal	1	0.98 (0.87–1.10)	1.01 (0.89–1.15)	1.14 (0.99–1.32)	0.174
Baked cereal	1	1.09 (0.97–1.23)	1.39 (1.22–1.58)	1.24 (1.08–1.43)	<0.001
Boiled cereal	1	0.98 (0.87–1.09)	1.01 (0.90–1.14)	1.09 (0.96–1.25)	0.153
Stir-fried cereal	1	0.96 (0.86–1.06)	1.02 (0.92–1.14)	0.95 (0.84–1.07)	0.588
**Excluding participants with extreme BMI (<18.5 and >40)**					
Total cereal	1	1.05 (0.94–1.16)	1.08 (0.96–1.21)	1.13 (1.00–1.28)	0.047
Fried cereal	1	0.97 (0.87–1.09)	1.03 (0.91–1.17)	1.15 (1.00–1.31)	0.093
Baked cereal	1	1.10 (0.98–1.23)	1.34 (1.19–1.52)	1.22 (1.07–1.40)	<0.001
Boiled cereal	1	0.98 (0.88–1.09)	1.04 (0.93–1.16)	1.10 (0.97–1.24)	0.093
Stir-fried cereal	1	0.93 (0.84–1.03)	1.02 (0.92–1.13)	0.97 (0.86–1.09)	0.787

HRs and 95% CIs were estimated using Cox proportional hazard regression models adjusted for age, sex, nationality (Han or non-Han), marital status (never married, married or living as married, widowed/divorced/separated, or unknown), BMI, household income (quintile), urbanization index, education (less than high school, high school, some college or at least college), physical activity (no regular activity, low to moderate activity or vigorous activity), smoking (never, former, current, or unknown), alcohol drinking status (abstainer or drinker) and medical insurance, total energy intake, vegetable intake, fruit intake, total meat intake, sodium intake, and potassium intake. CI, confidence interval; HR, Hazard ratio.
